# Self-organized nanocrystal rings formed by microemulsion for selective recognition of proteins and immunoassays†

**DOI:** 10.1039/c8ra09662g

**Published:** 2019-01-04

**Authors:** Jing Liang, Lei Yu, Ziying Lin, Keji Song, Jiejing Zhang, Jianfeng Zhang

**Affiliations:** College of Life Science, Jilin Agricultural University, Jilin Province Innovation Platform of Straw Comprehensive Utilization Technology Changchun 130118 China zhangjianfeng06@tsinghua.org.cn; Jilin Radio and TV University Changchun 130022 China

## Abstract

A simple and cheap method to fabricate a nanocrystal ring pattern was developed by utilization of a microemulsion in this study. The mixture of polystyrene and stabilizer dichloromethane solution that contained nanocrystal aqueous solution, prepared through shaking, was applied to fabricate a reverse microemulsion. After spreading and evaporating the solvent of microemulsion on a glass slide, an ordered honeycomb film was produced, accompanied by the formation of a nanocrystal ring pattern. The nanocrystal pattern could be readily applied for immunoassays and recognition of proteins. The pattern with antibody marked by a green colored nanocrystal specifically bound with antigen labeled by a red colored nanocrystal, leading to the enhancement in red fluorescent ring pattern and decrease in green fluorescent pattern. When the unlabeled antigen was added, the green fluorescent pattern was recovered. In addition, the ring pattern with immunocomplex could selectively recognize antigen and transferrin proteins. This strategy reveals that these patterns have potential applications in biochips, biosensors, imaging analysis and so forth.

## Abstract 2

Ordered nanocrystal rings were conveniently prepared by a microemulsion, which can further be applied for the recognition of proteins and immunoassays.

## Graphical abstract

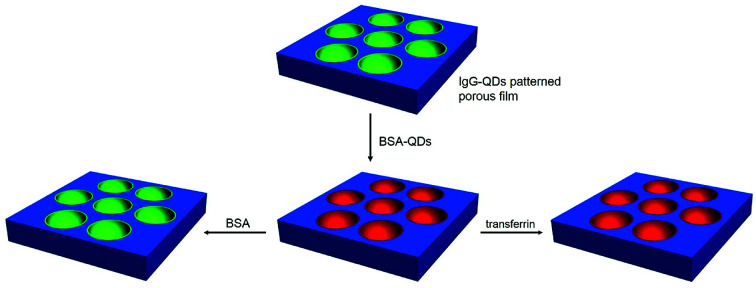

## Introduction

Nanocrystals (Quantum Dots, QDs) show unique electronic and optical properties and good biocompatibility, which make these materials attractive for many potential applications including energy storage, catalysis and biosensors.^1–4^ Patterning of QDs into well-defined micro/nanoscale architecture displays potentials in multiple color light-emitting diodes, color pixels for field-emission displays, multichannel chemical sensors, optical waveguides, and so on.^5^ In particular, the ring structures of QDs have attracted significant attention due to their applications as optical^6,7^ and electronic resonators.^8–10^ Several techniques have been developed for the formation of ring structures, such as photolithography,^11^ electron beam lithography,^12–14^ and molecular-beam epitaxy.^15,16^ However, the abovementioned methods face several limitations, such as inconvenience, high cost, and low throughput. Thus, efforts towards developing techniques avoiding the disadvantages are welcome.

Recently, a series of self-assembly techniques possessing low-cost features have been experimented to fabricate nanoparticle patterns.^17–19^ Among the various methods, breath figure, which uses water droplets as templates, has been widely investigated owing to its fast and easy operational features.^20,21^ CdSe nanoparticles decorating the walls of the holes on a polymer film^22^ and the CdS and ZnS QDs embedded in the porous film^23,24^ as well as silver nanoparticles with CdSe QDs located in the honeycomb architectures^25^ have been reported. Usually, the structures are obtained by the mixture of polymer and organic soluble QDs. However, these nanoparticles in the present form make the film preparation process complicated, which leads to an increase in difficulty in making efforts to achieve ordered patterns. In addition, the QDs pattern normally has random size, spacing, and periodicity. Moreover, the further applications of QDs pattern are less reported. Thus, overcoming these deficiencies and developing a convenient approach for preparing an ordered QDs ring pattern with further useful applications are essential. Recently, a novel way to synthesize ordered porous structures using microemulsion droplets as template has been proposed.^26,27^ Water-phase additives are located at the inner walls of the cavities, which are suitable for further modification of the pore surface. This strategy maintains the advantages of the breath figure method, while QDs pattern can be prepared in one step during the formation of a porous film.

**Scheme 1:**
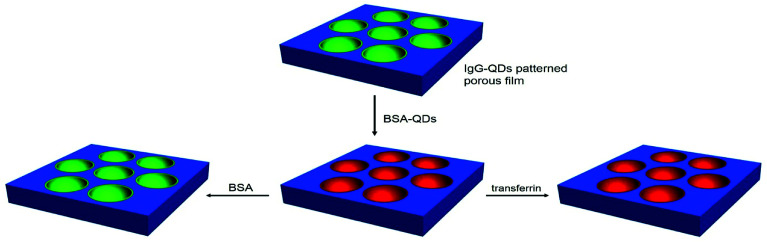
The schematic of assembly of QDs and *in situ* recognition of BSA and transferrin.

Herein, the QDs ring pattern has been prepared by a microemulsion method through the incorporation of QDs into the cavities on the polymer film. This strategy exhibits some advantages. The QDs are locally assembled in the cavities in one step, accompanied by the formation of the QDs ring structure. The QDs ring patterns have adjustable dimensions and high order, and their optical features can be maintained through a series of treatments in the porous film preparation process. Interestingly, the QDs ring pattern can be used to selectively recognize bovine serum albumin (BSA) and transferrin, and realize antigen–antibody immunoassays (Scheme 1). It should be noted that the procedures for the preparation of QDs ring pattern are convenient, low cost, and easy to operate. This reveals that these patterns have potential applications in biochip, microreaction technology, pattern recognition, biosensors, and cell culture.

## Experimental section

### Materials

Polystyrene serving as a film matrix (PS, molecular weight: 349 kg mol^−1^) was obtained from Sigma-Aldrich, USA. Didodecylamine (DDA), which was used as the stabilizer of water droplets, and *N*-hydroxysulfo-succinimide (NHS) were purchased from Aladdin, China. The coupling agent 1-(3-dimethylaminopropyl)-3-ethylcarbodiimide hydrochloride (EDC) was purchased from J&K Scientific Ltd, China. Transferrin and BSA were the products supplied by Beijing Biosynthesis Biotechnology Co. Ltd, China. Anti-BSA antibody (IgG) was the product supplied by Invitrogen, USA. The mercaptopropionic acid-modified CdTe (QDs) nanoparticles were synthesized according to the literature in this lab.^28^ The BSA-QDs and IgG-QDs were prepared through the procedures from the literature.^29^

### Preparation of nanocrystal ring pattern

The mixture of PS (6 mg mL^−1^) and DDA (0.6 mg mL^−1^) was prepared by simply adding them into a certain volume of dichloromethane. For the preparation of the microemulsion solution, a typical procedure was performed as follows: to the dichloromethane solution of PS and DDA, an aqueous QDs solution (0.5 mM) with green or red emission was added, and the volume fraction of water phase was maintained at 5% unless otherwise mentioned. The mixture solution was shaken for 30 s at 25 °C to disperse the aqueous solution in organic phase and achieve a translucent gray microemulsion. Then, 20 μL of the microemulsion solution was cast onto a glass slide under the relative humidity of 30–40% at the temperature of 25 °C to achieve QDs/PS porous films. Following the similar procedure, IgG-QDs/PS films were prepared.

### Immunoassays and recognition of BSA and transferrin

For the immunoassay experiment, as a general procedure, the IgG-QDs/PS porous film with green emission was first soaked into the BSA-QDs with red emission in pH = 7 PBS buffer solution for 12 h, followed by washing with water three times and drying in air. Then, the PS porous film patterned by the immunocomplex was immersed into the BSA (2 mM) buffer solution for 5 h, followed by washing with water three times and drying in air. For the recognition of proteins, the as-prepared PS porous film with immunocomplex was dipped into the transferrin buffer solution, followed by washing with water three times and drying in air.

### Measurements

Scanning electron microscopy (SEM) images were collected on a JEOL JSM-6700F field emission scanning electron microscope. Confocal laser scanning microscopic (CLSM) images were obtained on an Olympus Fluoview FV1000. Analysis of CLSM data was performed using the software FV10-ASW.

## Results and discussion

### Preparation and structural characterization of porous film

**Fig. 1:**
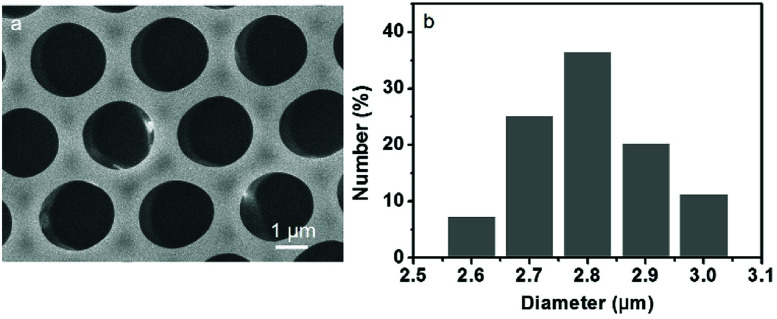
SEM images viewed from the (a) top surface and (b) histogram referring to the size distribution of cavities of the porous film prepared by casting the microemulsion solution on a glass slide.

Based on a similar principle to the breath figure for the preparation of ordered porous patterns on polymer surfaces, a different route for the fabrication of QDs patterned polymer films using a microemulsion was applied. As a typical strategy, the microemulsion was prepared through mixing of PS dichloromethane solution bearing DDA and an aqueous solution of CdTe QDs, followed by slight shaking. As a result, the reversed microemulsion droplets disperse in the organic solution evenly. After spreading the microemulsion solution on a glass slide, followed by the evaporation of the solvent, the porous polymer film was achieved under a certain temperature and humidity. The obtained polymer film exhibits bright iridescent colors when viewed along the reflection light, indicating a periodic refractive index variation with respect to the film thickness. The morphology of surface structure on the QDs/PS film was characterized through SEM measurements. The SEM image in Fig. 1a exhibits a highly ordered honeycomb-patterned film showing monodispersed hexagonal close-packed cavities. The histogram (Fig. 1b) illustrates that the diameter of the cavities is about 2.8 μm.

### The characterization of QDs ring structure

**Fig. 2:**
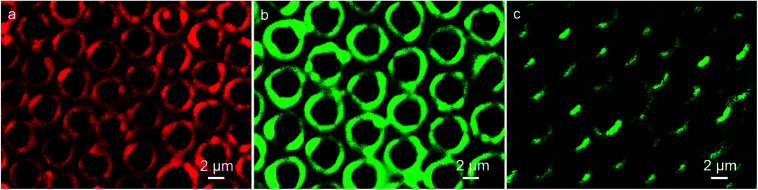
CLSM images of PS porous film with (a) red and (b) green fluorescent QDs, (c) without QDs additives after immersion into the green fluorescent QDs aqueous solution.

In the microemulsion approach, the additives that were pre-fixed into the water phase were located at the inner walls of the cavities after the formation of the porous film. Herein, the CdTe QDs aqueous solution was applied as an additive to enhance the functionality of the porous structures. The emission spectra of the green and red fluorescent QDs are shown in Fig. S1,† revealing the luminescent features of the as-prepared QDs. Due to the ionic interaction, the QDs additives initially added in water droplets deposit on the inner walls of cavities, where the stabilizer DDA is located. As presented in Fig. 2a and b, discrete fluorescent rings from QDs with red and green emission in patterned arrays indicate that the QDs anchor on the inner walls of the voids, accompanied by the formation of porous surface. Thus, the red and green color nanocrystal ring patterns were prepared in one step. However, when the PS porous film was prepared without the QDs additives in the fabrication process of microemulsion and followed with the immersion into the CdTe QDs aqueous solution, very weak fluorescent ring structures were observed (Fig. 2c). This indicates the efficiency of the present method for the preparation of the nanocrystal ring structure, which avoids the complicated procedures and guarantees the regularity of the pattern. In the microemulsion method, CdTe QDs behave only as additives, which implies that almost all the water-soluble but organic-insoluble QDs interacting with stabilizer are applicable to be incorporated into the patterned cavities, resulting in the formation of ring patterns.

### Immunoassays and recognition of proteins

**Fig. 3:**
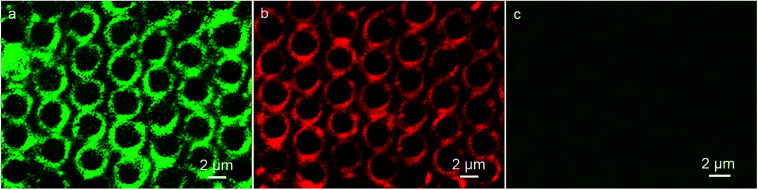
CLSM images of IgG-QDs ring pattern before (a) and after encountering the immersion in BSA-QDs solution observed in (b) red and (c) green fluorescent modes.

**Fig. 4:**
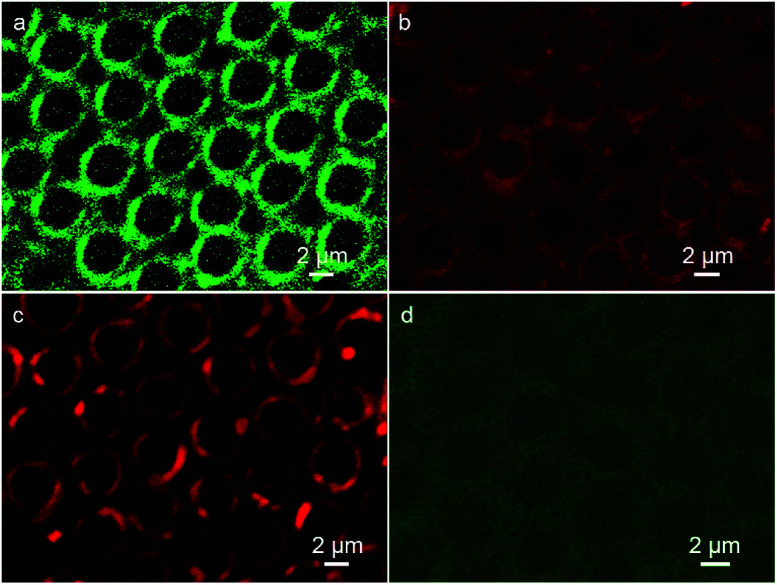
CLSM images of ring pattern with immunocomplex after immersion in the (a and b) unlabeled BSA solution observed in green and red fluorescent modes, and (c and d) transferrin solution observed in red and green fluorescent modes, respectively.

Immunoassay plays a crucial role in clinical, pharmaceutical and environmental chemistry. Among the immunoassays, the assay based on antigen–antibody binding is widely used. The excitation band of QDs is rather wide, while the emission spectra is quite narrow due to their quantum confinement effects, which leads to the wavelengths to be tunable over a wide range, with the change in particle size. The result of absorption spectroscopy and transmission electron microscopy (TEM) shows the numerical value of wavelengths and particle sizes of the CdTe QDs, respectively, (Fig. S2 and S3†). The green fluorescent QDs correspond to the wavelength of 482 nm and dimension of about 2.8 nm, while the red color QDs show emission at 587 nm and have size of about 3.9 nm, which are in accordance with the reported calculation method in the literature.^30^ Thus, the immunoassays based on the different imaging color of the same QDs material with different dimension using the same excitation source are available. Herein, the detection which can be realized on the interface and surface, makes the analysis become more sensitive. Typically, we first synthesized the IgG-QDs with green color, and their emission spectrum indicated that the optical properties of QDs were retained after coupling and conjugation (Fig. S1c†). Then, the IgG-QDs as water-phase additives were incorporated into the cavities. As presented in Fig. 3a, similar to the result of pure CdTe QDs pattern, discrete fluorescent rings of IgG-QDs on honeycomb patterned arrays are observed. When the IgG-QDs pattern is dipped into BSA-QDs buffer solution with red emission, the CLSM image illustrates that the strong red fluorescent pattern of BSA-QDs emerges at the position where the IgG-QDs are located (Fig. 3b) and the corresponding green fluorescent pattern of IgG-QDs is quenched (Fig. 3c). This phenomenon is attributed to the binding of antigen and antibody that brings the two QDs close enough to allow the occurrence of Förster resonance energy transfer (FRET). Thus, the energy of the excitonic state in the QDs with green color is transferred to the similar state of the QDs with red color carrying lower exciton energy. The efficiency of this FRET process is high due to the strong overlap of the emission and absorption spectra of green and red QDs pairs. Interestingly, when the ring pattern with immunocomplex was immersed into the unlabeled BSA buffer solution, green fluorescent pattern from the IgG-QDs is recovered, accompanied by the decrease in red fluorescence from the BSA-QDs, as shown in Fig. 4a and b. The reason is that the unlabeled BSA competitively binds to IgG-QDs, thus replacing red fluorescent-marked BSA in the immunocomplex and inhibiting the FRET process. Thus, the green fluorescent ring pattern can be observed again, while the red fluorescent pattern weakens. This strategy provides an alternative route to realize immunoassays based on a highly visual imaging analysis.

To examine the selective recognition ability of the nanocrystal ring pattern, transferrin was selected as a protein model. When the patterned film decorated with the immunocomplex was immersed into the transferrin solution, the color of the pattern does not change (Fig. 4c and d). Transferrin, which does not bind to antibody IgG-QDs, reveals that there is no influence on the FRET process between BSA-QDs and IgG-QDs. On the contrary, when the ring pattern with immunocomplex was dipped into the BSA solution, the competitive combination appears, as shown in Fig. 4a and b, displaying the occurrence of FRET process. This fact demonstrates the high biospecificity of the as-prepared ring pattern with immunocomplex, which will not be interfered by other proteins.

## Conclusion

In conclusion, the nanocrystal ring patterns have been developed by the utilization of a microemulsion approach through the one-step assembly of QDs into the cavities of the porous film. The process for the preparation of nanocrystal ring patterns is much simpler, more facile, cheaper, saves some complicated steps and avoids expensive instruments and operations, which are applied in other techniques for pattern formation. Interestingly, the excellent optical features of the QDs are maintained. Furthermore, the QDs ring pattern, which realizes the specific immunoassays and selectively recognizes BSA and transferrin, proved to have potential applications on biochips and imaging analysis. It can be envisioned that this method can be applicable to other immunoassay systems or biological specific binding systems, which further endow the pattern with a variety of potential applications in biosensors, biomedicines, and so forth.

## Conflicts of interest

There are no conflicts to declare.

## Supplementary Material

RA-009-C8RA09662G-s001
