# Supplementary material for: Self-organized nanocrystal rings formed by microemulsion for selective recognition of proteins and immunoassays
Source: RSC Adv. 2019 Jan 4;9(2):699–703. doi: 10.1039/c8ra09662g (PMC9059482; doi:10.1039/c8ra09662g)
Supplement: RA-009-C8RA09662G-s001 [file RA-009-C8RA09662G-s001.pdf]

Electronic Supplementary Material (ESI) for RSC Advances.
This journal is © The Royal Society of Chemistry 2018

Supplementary Information

Self-organized Nanocrystal Rings Formed by Microemulsion for Selective Recognition of Proteins and Immunoassays

Jing Liang,[a] Lei Yu,[b] Ziying Lin,[a] Keji Song,[a] Jiejing Zhang,[a] Jianfeng Zhang*[a]

[a] College of life science, Jilin Agricultural University, Changchun 130118, China

[b] Jilin Radion and TV University, Changchun 130022, China

E-mail: zhangjianfeng06@tsinghua.org.cn

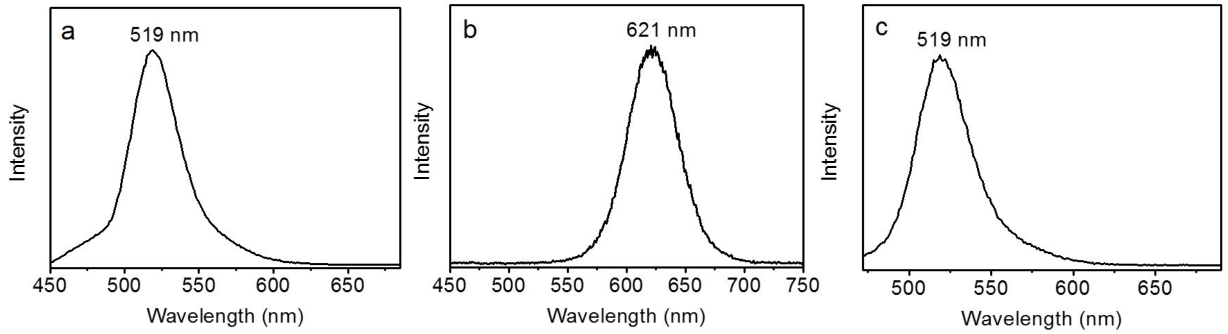

Fig. S1 Emission spectra of QDs with (a) green and (b) red color fluorescence, (c) QDs-IgG with green fluorescence, respectively.

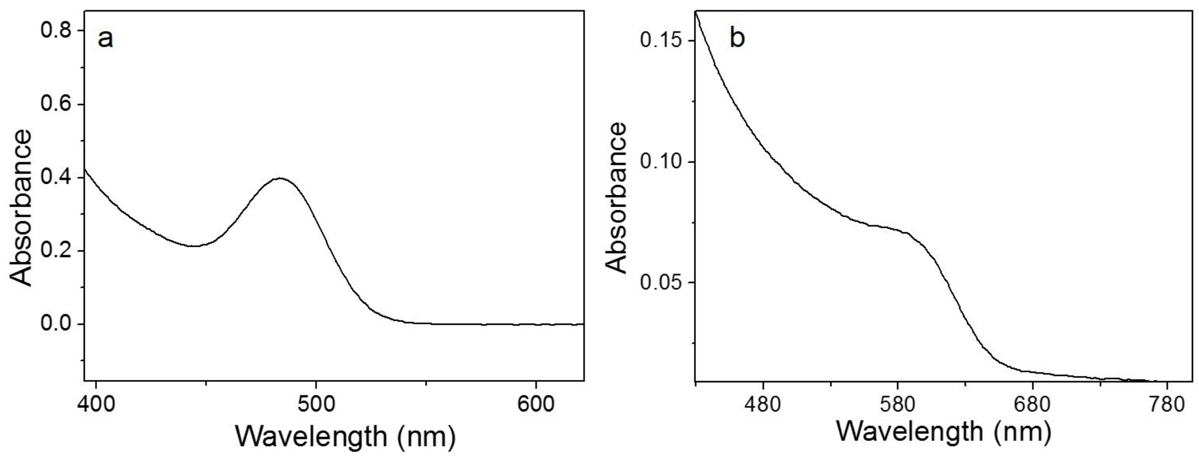

Fig. S2 Absorption spectra of CdTe QDs with (a) green and (b) red color fluorescence, respectively.

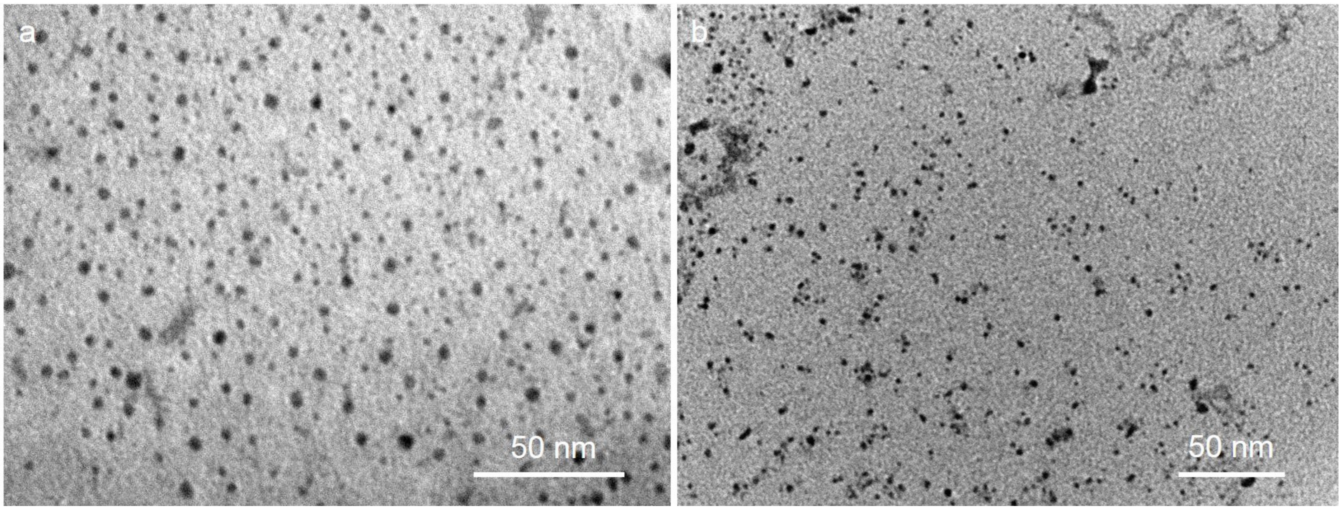

Fig. S3 TEM images of CdTe QDs with (a) green and (b) red color fluorescence, respectively.
